# Titanium–Tissue Interface Reaction and Its Control With Surface Treatment

**DOI:** 10.3389/fbioe.2019.00170

**Published:** 2019-07-17

**Authors:** Takao Hanawa

**Affiliations:** Department of Metallic Biomaterials, Institute of Biomaterials and Bioengineering, Tokyo Medical and Dental University, Tokyo, Japan

**Keywords:** titanium, titanium alloy, biocompatibility, biofunction, bone formation, bone bonding, surface treatment, surface morphology

## Abstract

Titanium (Ti) and its alloys are widely used for medical and dental implant devices—artificial joints, bone fixators, spinal fixators, dental implant, etc. —because they show excellent corrosion resistance and good hard-tissue compatibility (bone formation and bone bonding ability). Osseointegration is the first requirement of the interface structure between titanium and bone tissue. This concept of osseointegration was immediately spread to dental-materials researchers worldwide to show the advantages of titanium as an implant material compared with other metals. Since the concept of osseointegration was developed, the cause of osseointegration has been actively investigated. The surface chemical state, adsorption characteristics of protein, and bone tissue formation process have also been evaluated. To accelerate osseointegration, roughened and porous surfaces are effective. HA and TiO_2_ coatings prepared by plasma spray and an electrochemical technique, as well as alkalinization of the surface, are also effective to improve hard-tissue compatibility. Various immobilization techniques for biofunctional molecules have been developed for bone formation and prevention of platelet and bacteria adhesion. These techniques make it possible to apply Ti to a scaffold of tissue engineering. The elucidation of the mechanism of the excellent biocompatibility of Ti can provide a shorter way to develop optimal surfaces. This review should enhance the understanding of the properties and biocompatibility of Ti and highlight the significance of surface treatment.

## Introduction

**Figure 1:**
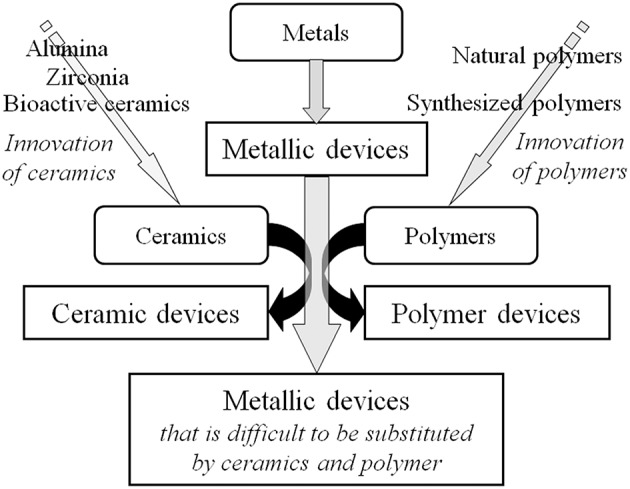
Substitution of metallic devices by ceramic devices and polymer devices due to innovation of ceramics and polymers.

Many medical devices made of metals have been substituted by those made of ceramics and polymers during the past half century because of innovation in ceramics and polymers and their excellent biocompatibility and biofunction, as shown in Figure 1. Despite this situation, more than 70% of surgical implant devices, especially more than 95% of orthopedic implants (calculated based on statics from the Ministry of Health, Labor and Welfare, Japan), still consist of metals because of the large fracture toughness and durability of metals. In particular, titanium (Ti) materials, such as commercially pure titanium (CP Ti) and Ti alloys are widely used in medicine and dentistry because of their large corrosion resistance, large specific strength, and high performance in medicine and dentistry (Brunette et al., 2001). Their good interfacial and chemical compatibility against tissues are well-known based on substantial evidence from basic research and high clinical performances. However, the mechanism of the excellent biocompatibility of Ti among metals is not completely understood. After a metallic material is implanted into a human body, a reaction immediately occurs between the living tissue and the material surface. In other words, the first reaction at the interface directly influences the material's biocompatibility. The Young's modulus of α + β-type Ti alloy (100–111 GPa) is half those of type 316L stainless steel (200 GPa) and Cobalt (Co)–chromium (Cr)–molybdenum (Mo) alloy (~220 GPa), which is a large advantage to prevent stress shielding in bone plates and stems of artificial hip joints in orthopedics. In addition, the magnetic susceptibilities of Ti (31.9 × 10^−9^ m^3^ kg^−1^) and Ti−6Al−4V ELI alloy (39.8 × 10^−9^ m^3^ kg^−1^) are much smaller than that of Co–Cr–Mo alloy (94.5 × 10^−9^ m^3^ kg^−1^), as well as stainless steels, decreasing the influences of magnetic resonance imaging (MRI), such as motion, attraction force, torque, heat generation, and artifacts. This property is significant, because MRI is commonly used for medical examination.

A disadvantage of metals for use as biomaterials is that they are artificial materials, and metals do not have biofunction. To promote biocompatibility and add biofunction to metals, surface modification or surface treatment is necessary, because biocompatibility is not promoted and biofunction is not added through conventional manufacturing processes, such as melting, casting, forging, and heat treatment. Surface treatment is a process that changes surface morphology, structure, and composition, leaving the bulk mechanical properties. In orthopedics, bone bonding is required in the stem and acetabular cup of artificial hip joints. In the case of dentistry, hard-tissue compatibility for bone formation and bone bonding, soft-tissue compatibility for adhesion of gingival epithelium, and an antibacterial property for the inhibition of bacterial invasion are required in dental implants. For these purposes, a variety of surface treatment techniques have been investigated at the research level, and some of them have been commercialized.

In this overview, a brief history of CP Ti and Ti alloys, the application of Ti to medical devices (including dental devices), their use and tasks in medicine, proposed mechanisms of excellent biocompatibility of Ti, and surface treatment to improve biocompatibility and to add biofunction are reviewed. This review is intended to enhance the understanding of the properties and biocompatibility of Ti and the significance of surface treatment, including surface-morphological alteration.

## History of Application to Medicine

The history of the application of CP Ti and Ti alloys to medicine and dentistry is summarized in Table 1. The first report on CP Ti for medicine was appeared in 1940, and excellent bone compatibility was found based on an animal test (Bothe et al., 1940). Thereafter, the compatibility to bone and soft tissue of rabbits (Leventhal, 1951), its non-cytotoxicity due to excellent corrosion resistance in biological environments (Beder et al., 1957), and excellent biocompatibility in dogs were reported. The large-scale industrial manufacturing process for Ti achieved in the last half 1940s made it possible to conduct many studies for medical applications, revealing excellent biocompatibility in long-term animal testing (Williams, 1982a). Thereafter, the usefulness of CP Ti was widely recognized by the last half 1960s through clinical evaluation (Pillar and Weatherly, 1982; Williams, 1982a,b).

**Table 1:** History of titanium application to medicine and development of titanium alloys.

**Year**	**Material**	**Circumstance**	**References**
1940	Ti	Confirmation of equivalent biocompatibility as stainless steel and cobalt-chromium alloy with animal test	Bothe et al., 1940
1940	Ti	Success of smelting by Kroll process	Kroll, 1940
1948	Ti	Launching industrial production	
1951	Ti	Confirmation of both soft and hard tissues compatibility with animal test	Leventhal, 1951
1957	Ti	Confirmation of non-toxicity with long-term implantation	Beder et al., 1957
1959	Ti–Ni	Development of shape memory alloy in USA	Buehler et al., 1963; Wang et al., 1965
1960	Ti	Excellent results in artificial joints	Williams, 1982a
1960's	Ti	Marketing as surgical implants in UK and USA	
1970's	Ti−6Al−4V	Diverting aircraft material to orthopedic implants	
1978	Ti–Cu–Ni	Trial of dental casting	Waterstrat et al., 1978
1980	Ti−5Al−2.5Fe	Development in Europe	
1982	Ti	Development of investment material and casting machine for dental casting	Miura and Ida, 1988
1985	Ti−6Al−7Nb	Development in Switzerland	Semlitsch and Staub, 1985
1993	Ti−13Nb−13Zr	Development in USA	
1993	Ti−12Mo−6Zr−2Fe	Development in USA	Wang et al., 1993
1996	Ti−15Mo	Development in USA	Zardiackas et al., 1996
1988	Ti−29Nb−13Ta−4.6Zr	Development in Japan	Kuroda et al., 1988
Around 2000	Ti−15Mo−5Zr−3Al	Development in Japan	Rao and Houska, 1979; Matsuda et al., 1997
Around 2000	Ti−6Al−2Nb−1Ta−0.8Mo	Development in Japan	Okazaki, 2001
2004	Ti−15Zr−4Nb−4Ta	Development in Japan	Ozaki et al., 2004
After 2000	β-metastable alloys based on TRIP and TWIP	Development in mainly China	Marteleur et al., 2012; Ahmed et al., 2016: Brozek et al., 2016; Zhan et al., 2016; Zhang et al., 2017; Lai et al., 2018

However, to avoid the fracture of CP Ti in the human body, an aerospace Ti−6Al−4V alloy was diverted to artificial joints and bone fixators (Pillar and Weatherly, 1982; Williams, 1982a,b). Thereafter, vanadium (V)- and/or aluminum (Al)-free α + β-type Ti alloys and β-type Ti alloys with low Young's modulus have been developed. V that creates the cytotoxicity of Ti−6Al−4V alloy was replaced by niobium (Nb), which is a safe element, to develop a new α + β-type Ti−6Al−7Nb alloy (Semlitsch and Staub, 1985; Li et al., 2010). Other α + β-type alloys, Ti−6Al−2.5iron (Fe) alloy and Ti−6Al−2Nb−1 tantalum (Ta)−0.8Mo alloy, were developed in 1970s (Rao and Houska, 1979; Anon, 1994).

On the other hand, β-type Ti alloys for medical use have been developed. Ti−13zirconium (Zr)−13Ta alloy (nearly β) has been developed in the United States. Various β-type alloys, Ti−12Mo−6Zr−2Fe alloys (Wang et al., 1993), T−15Mo (Zardiackas et al., 1996), and Ti−15Mo−2.8Nb−0.2silicon (Si)−0.28oxygen (O) (Fanning, 1996), have been developed in the United States. Ti−15Mo−5Zr and Ti−15Mo−5Zr−3Al alloys (Rao and Houska, 1979; Matsuda et al., 1997) and Ti−15Zr−4Nb−4Ta alloy (Okazaki, 2001) have been developed in Japan. The history of the development of β-type Ti alloys is well-summarized elsewhere (Niinomi, 2019). Young's modulus could decrease to 40–60 GPa in a β-type alloy.

Since 2000, a new wave of the development of Ti alloys has been generated. The design of Ti alloys through twinning-induced plasticity (TWIP) and transformation-induced plasticity (TRIP) has been attempted, making it possible to develop novel β-metastable Ti alloys (Marteleur et al., 2012; Ahmed et al., 2016: Brozek et al., 2016; Zhan et al., 2016; Zhang et al., 2017; Lai et al., 2018). The TRIP and TWIP concepts were first invented in the field of steels and applied to Ti alloys through Ti–nickel (Ni) shape memory alloy. It is possible that this design will be applied to biomedical alloys in the near future.

In dentistry, CP Ti has been successfully used for dental implants since 1965 (Waterstrat et al., 1978), and the excellent hard-tissue compatibility is well-known. A magnesia-system investment material and argon-arc casting machine were developed in 1982, followed by the development of various dental casting systems for dental restoratives (Miura and Ida, 1988).

The development of new Ti alloys for medical devices continuously challenges by researchers, and new designs have been attempted based on d-electron alloy design theory (Kuroda et al., 1988) and the TRIP and TWIP concept.

## Medical Application and Tasks of Titanium

Because of the excellent properties of CP Ti and Ti alloy as biomaterials, they are used for devices requiring strength, elongation, and long-term bone bonding in orthopedics, cardiovascular medicine, dentistry, etc. The specifications of Ti alloys used for medicine are listed in Table 2. Medical devices and CP Ti and Ti alloys are listed in Table 3, and problems of CP Ti and Ti alloys in medicine are summarized in Table 4.

**Table 2:** Specification of titanium alloys for medical use.

**Composition (mass%)**	**Type**	**ASTM**	**ISO**	**JIS**
Ti−5Al−2.5Fe	α + β	–	ISO 5832-10	
Ti−6Al−4V	α + β	F1108 (Cast) F1472 (Wrought)	ISO 5832-3	T7401-2
Ti−6Al−4V ELI	α + β	F136 (Wrought)	ISO 5832-3	–
Ti−6Al−2Nb−1Ta	α + β	–	–	T7401-3
T−15Zr−4Nb−4Ta	α + β	–	–	T7401-4
Ti−6Al−7Nb	α + β	F1295	ISO 5832-11	T7401-5
Ti−3Al−2.5V	α + β	F2146		
Ti−6Al−2Nb−1Ta−0.8Mo	α + β	F136	ISO 5832-14	
Ti−13Nb−13Zr	Near β	F1713	–	
Ti−15Mo	β	F2066	–	
Ti−12Mo−6Zr−2Fe	β	F1813	–	
Ti−15Mo−5Zr−3Al	β	F136	ISO 5832-14	T7401-6
Ti−55.8Ni	Intermetallic compound	ASTM F 2063		T7404

**Table 3:** Medical devices consisting of titanium and titanium alloys.

**Clinical department**	**Medical device**	**CP Ti and Ti alloy**
Orthopedics	Spinal fixator	CP Ti; Ti−6Al−4V; Ti−6Al−7Nb
	Bone fixator (bone plate, screw, wire, bone nail, mini palate, etc.)	CP Ti; Ti−6Al−4V; Ti−6Al−7Nb
	Artificial joint; artificial head	Ti−6Al−4V; Ti−6Al−7Nb; Ti−15Mo−5Zr−3Al; Ti−6Al−2Nb−1Ta−0.8Mo
	Spinal spacer	Ti−6Al−4V; Ti−6Al−7Nb
Cardiovascular department	Implantable artificial heart (housing)	CP Ti
	Heart pacemaker (case) (electrode) (terminal)	CP Ti; Ti−6Al−4V CP Ti CP Ti
	Artificial valve (flame)	Ti−6Al−4V
	Vascular stent	Ti–Ni
	Guide wire	Ti–Ni
	Cerebral aneurysm clip	CP Ti; Ti−6Al−4V
Dentistry	Inlay; crown; bridge; clasp; denture base	CP Ti; Ti−6Al−7Nb
	Dental implant	CP Ti; Ti−6Al−4V; Ti−6Al−7Nb
	Orthodontic wire	Ti–Ni; Ti–Mo
General surgery	Surgical instrument (scalpel; tweezer; scissor; drill)	CP Ti
	Catheter	Ti–Ni

**Table 4:** Problem to be solved in titanium and titanium alloys for medical use.

**Problem**	**Material**	**Medical device**
Stress shielding	α + β type Ti alloy	Bone plate; stem of artificial hip joint
Adhesion to bone	Whole Ti alloy	Bone screw; bone nail
Cracking and fracture by excessive deformation	CP Ti, α + β type Ti alloy	Spinal rod; maxillofacial plate
Crevice corrosion; pitting	Ti–Ni alloy	Stent graft
Fracture	Ti–Ni alloy	Endodontic file
Corrosion with fluoride	CP Ti; whole Ti alloy	Dental restorative
Cytotoxicity	CP Ti; whole Ti alloy	All devices
Peri-implantitis	CP Ti; whole Ti alloy	Abutment of dental implant; orthodontic implant anchor; percutaneous device; screw of external bone fixator

Ti alloys are used in orthopedics for artificial joints, bone fixators, spinal fixators, etc., receiving large mechanical stress. Bone absorption caused by stress shielding sometimes appears in bone fixators and artificial hip joints. Because load is mainly applied to the metal plate and stem, less load is applied to cortical bone by the difference in Young's modulus between metal and cortical bone (Gefen, 2002). If the Young's modulus of the metal plate is similar to that of cortical bone, load is equally applied to both metal and bone to prevent bone absorption. In this sense, β-type Ti alloys showing a lower Young's modulus are more suitable than α + β-type alloys. Therefore, β-type Ti alloys consisting of Group 4 and 5 elements in the periodic table have continued to be designed and developed.

However, bone screws and bone nails made of Ti alloys form calluses and assimilate to bone tissue, forming calluses, during implantation, so bone is sometimes refractured when the devices are retrieved (Sanderson et al., 1992). Therefore, when the devices must be retrieved after healing, devices made of 316L-type stainless steel are selected. This assimilation occurs because of the excellent hard-tissue compatibility of Ti alloys. A proper surface treatment may inhibit bone formation and bonding of Ti alloys contacting bone tissue.

In spinal surgery and maxillofacial surgery, the rod and plate of Ti alloys are sometimes bent by medical doctors in the operation room. These operations sometimes generate crack or fracture of Ti alloys, because the elongation to fracture of α + β-type Ti alloy (10% of Ti−6Al−4V ELI; Brunette et al., 2001) is much smaller than that of 316L-type stainless steel (40%; ASTM A240). Therefore, the strengthening of α + β-type Ti alloy while maintaining elongation is required.

Ti–Ni alloy is used as guidewires and self-expanding stents. However, 37.2% (45 of 121 cases) of Ti–Ni stents are fractured in 10.7 months of service (Scheinert et al., 2005). Corrosion may be related to the fracture, while the main cause is fatigue. In the case of stent grafts of Ti–Ni, severe pitting and crevice corrosion appears by the acceleration of corrosion due to the crevice between Ti–Ni alloy and a polymer as an artificial blood vessel (Heintz et al., 2001). Therefore, Ni-free Ti-based superelastic alloys have been researched (Shinohara et al., 2015).

In dentistry, the fixture part of dental implants consists of CP Ti and Ti alloys to bond alveolar bone. A Ti–Ni superelastic alloy and a Ti–Mo alloy are used as orthodontic arch wire. In particular, Ti–Ni alloy is widely used, because proper and continuous orthodontic force remains for a long time. Ti–Ni alloy is suitable for reamers and files for endodontics for bending tooth roots, while the alloy sometimes fractures from an overload with dental engines.

Corrosion of metallic implant devices implanted into the human body has been studied (Nakayama et al., 1989; Brunette et al., 2001; Alves et al., 2009; Asri et al., 2017; Manam et al., 2017; Eliaz, 2019), because the corrosion is related to toxicity and fracture, whereas examples of corrosion-fracture of metal implants are few. The reason is because the retrieval case of implants is limited, and surgeons are rarely interested in corroded retrieved implants. In particular, severe corrosion cases of CP Ti and Ti alloys are rare. However, Ti used as dental restoratives is corroded by fluorine compounds contained in mouthwashes and dental pastes (Nakagawa et al., 1999). Microbial corrosion of Ti in the oral cavity has also been studied (Fukushima et al., 2014). The corrosion phenomena of metallic biomaterials including Ti alloys are reviewed (Manam et al., 2017; Eliaz, 2019), while the case of Ti alloys is rare.

As described above, CP Ti and Ti alloys are widely used in medicine and dentistry because of their lightness, high corrosion resistance, and excellent biocompatibility compared with other metals.

## Biocompatibility of Titanium

**Figure 2:**
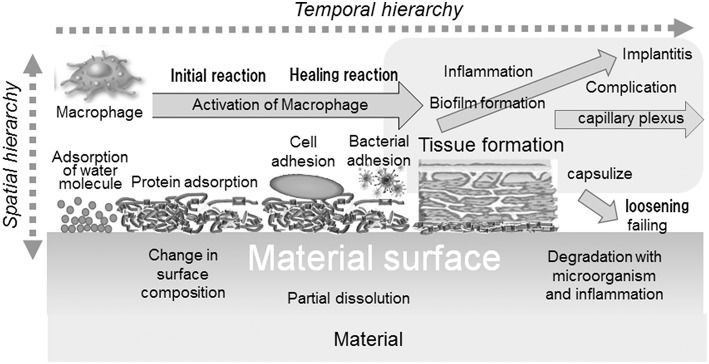
Interfacial reactions of materials and the host body.

Biocompatibility is defined as “the ability of a material to perform with an appropriate host response in a specific application” (William, 1987). The biocompatibility of a material is governed by initial and continuous reactions between the material and host body: adsorption of molecules, protein adsorption, cell adhesion, bacterial adhesion, activation of macrophage, formation of tissues, inflammation, etc. In addition, the reaction occurs with a temporal and spatial hierarchy, as illustrated in Figure 2.

CP Ti shows a unique property, “osseointegration,” among metals. Osseointegration is defined as follows. It is the “formation of a direct interface between an implant and bone, without intervening soft tissue. No scar tissue, cartilage or ligament fibers are present between the bone and implant surface. The direct contact of bone and implant surface can be verified microscopically” (Brånemark et al., 1977). Osseointegration shows the excellent hard-tissue property of Ti. This concept, osseointegration, in dental implants generated and explosively accelerated studies on the reaction between hard tissue (bone and tooth) and Ti, followed by studies on surface treatment.

Extensive research on the hard-tissue compatibility of Ti has been reported; it is impossible to introduce everything here, so we advise referring to a book in which it is reviewed (Brunette et al., 2001). Excellent hard-tissue compatibility of Ti was confirmed by studies on calcium phosphate formation ability in simulated body fluids; evaluation of osteoblast activity and calcification; histological and molecular-biological evaluation of Ti implanted in animals, such as bone formation, bone contacting rate, and bone bonding strength; and clinical results. The above results revealed that, when Ti is implanted in bone, the surrounding tissue contacts Ti in an early stage, and the bone bonding strength is large. Important factors governing hard-tissue compatibility are the adhesion and proliferation of osteogenic cells because of the surface morphology (roughness), wettability, etc. Bone formation occurs through the inflammatory response period, osteoblast induction period, and bone formation period. The surfaces of Ti implant and Ti–bone interface reaction have been characterized to explain the importance of surface morphology, wettability, and energy for osseointegration (Rupp et al., 2018; Shah et al., 2018, 2019). The surface of Ti implants stored for a long time after manufacturing becomes contaminated, and the bone conduction ability is depressed during storage (Art et al., 2009).

Bonding between metals and soft tissue is also important in abutments of dental implants, orthodontic implant anchors, transdermal devices, and screws of external fixators. In these devices, metals penetrate from the inside to the outside of tissues. Therefore, insufficient bonding of soft tissue makes the invasion of bacteria that generates inflammation possible, followed by loosening, movement, and falling out of the implant. In the case of dental implants, these events are known as peri-implantitis. Other medical devices completely implanted in tissues may be covered by fibrous tissue unless enough soft-tissue compatibility is shown. It is well-known that Ti shows good soft-tissue compatibility only in the case of complete implantation, while chemical bonding of soft tissue to Ti is not observed. In particular, despite the significance of the adhesion of junctional epithelium to Ti in dental implants, this subject is still unresolved. Bonding of junctional epithelium to Ti is attempted by a mechanical anchoring with rough or grooved Ti surfaces at present, because chemical adhesion of soft tissue to metals is difficult (Williams, 2011).

A platelet adhesion test with human blood revealed that platelets easily adhered and a fibrin network formed on Ti (Tanaka et al., 2009; Ratner et al., 2013). Ti may form a thrombus easily and show low blood compatibility. Probably for this reason, bare Ti and Ti alloys except Ti-Ni alloy are not used for devices contacting blood.

## Mechanism of Biocompatibility of Titanium

### Response of the Host Body

The interface between Ti and bone tissue has been observed from early on at a micrometer and nanometer scale (Albrektsson and Hansson, 1986; Davies et al., 1990; Listgarten et al., 1992; Sennerby et al., 1993; Murai et al., 1996; Branemark et al., 1998; Sundell et al., 2017). Metal Ti substrate is covered by titanium oxide (a few nanometers in thickness), an amorphous layer containing proteoglycans (20–50 nm in thickness), a slender cell layer, a weakly calcified region, and bone tissue, in that order. Endeavors to observe a structure near the Ti surface have continued to elucidate the mechanism of osseointegration (Palmquist et al., 2010; Goriainov et al., 2014).

Recently, red-blood-cell and platelet interactions (Park and Davies, 2000), wettability and hydrophilicity (Gittens et al., 2014; Albrektsson and Wennerberg, 2019), increase in osteogenesis-, angiogenesis-, and neurogenesis-associated gene expression (Salvi et al., 2015), healing- and immune-modulating effect (Trindade et al., 2016), immune osteocyte-related molecular signaling mechanisms (Shah et al., 2018), and inflammation-immunological balance (Trindade et al., 2018; Albrektsson et al., 2019) have been considered as factors of osseointegration.

However, the focus of the research moved to surface treatments to accelerate bone formation and bone bonding. The reaction mechanism is usually investigated to explain the effect of the treatments. The above phenomena are caused by the surface properties of Ti and situational evidence; the surface properties causing the above phenomena must be understood. Properties of the Ti surface that may cause osseointegration are explained in the following subsections.

### Corrosion Resistance

**Figure 3:**
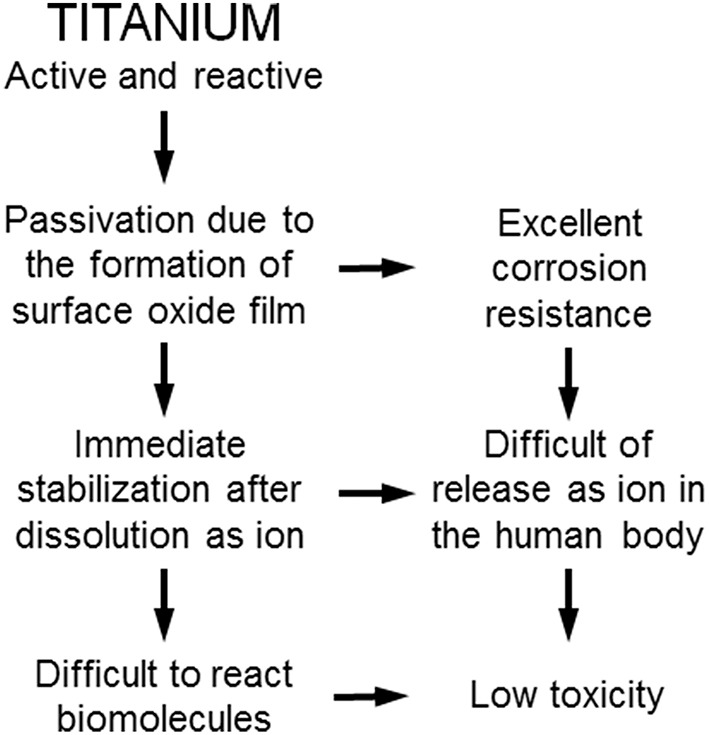
Excellent corrosion resistance and low toxicity of titanium based on its high activity.

**Figure 4:**
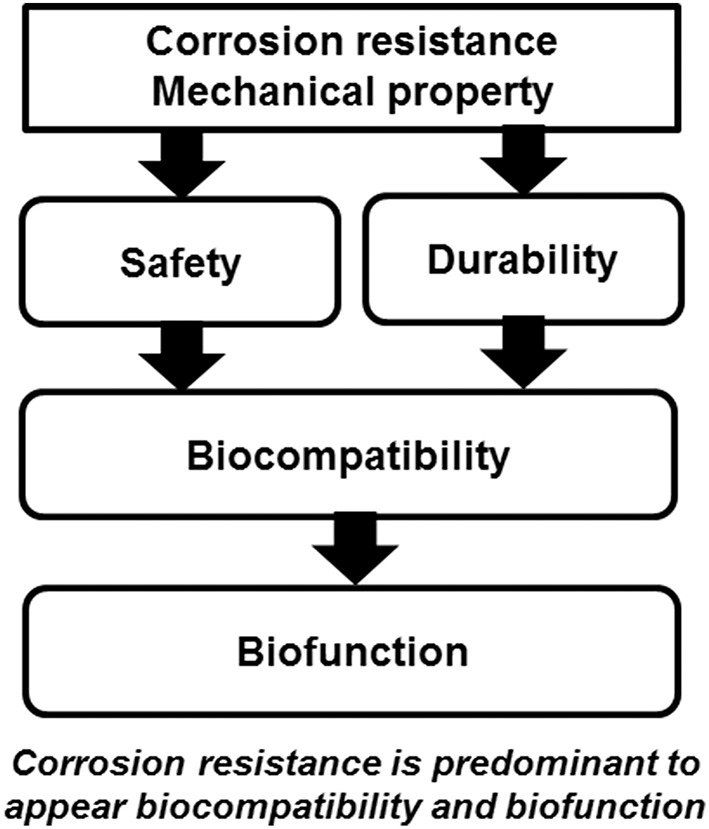
Biocompatibility and biofunction based on corrosion resistance and mechanical property.

Ti shows excellent corrosion resistance compared with other metals (Nakayama et al., 1989; Brunette et al., 2001; Asri et al., 2017; Manam et al., 2017; Eliaz, 2019), inducing low toxicity (Figure 3). One of the reasons for the excellent biocompatibility of Ti is caused by the excellent corrosion resistance, while the corrosion resistance is not sufficient condition for the biocompatibility. Even the best corrosion-resistant metal, Au, is inferior in tissue compatibility. In addition, electric plating of Pt to Ti increases the corrosion resistance but depletes bone formation (Itakura et al., 1989), because a property of Ti is shielded, and the bone formation ability is prevented. These results reveal that hard-tissue compatibility is not induced only by the corrosion resistance. In other words, the corrosion resistance is a necessary condition but not a sufficient condition for biocompatibility; there are other factors that contribute to biocompatibility. This concept is illustrated in Figure 4.

### Surface Hydroxyl Groups

**Figure 5:**
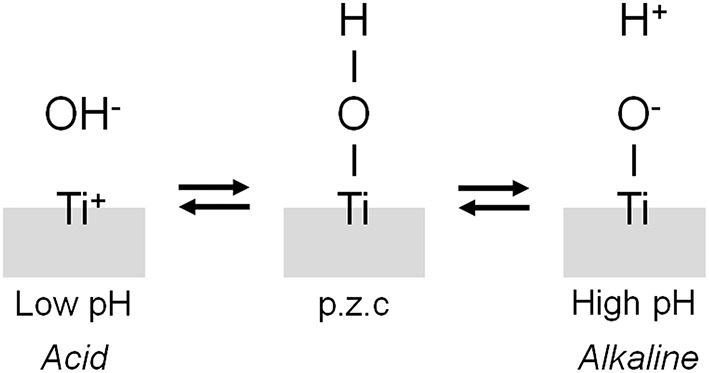
Dissociation of surface hydroxyl group on metal.

The interface reaction between Ti and living tissue is governed by the property of surface oxide film (passive film) covering the Ti substrate. This surface oxide film forms hydroxyl groups on itself because of a reaction with moisture in the air (Boehm, 1966). These hydroxyl groups dissociate in aqueous solutions, such as body fluid, to form electric charges (Boehm, 1966, 1971; Parfitt, 1976). The electric charge depends on the pH of the surrounding solution, and it becomes zero at a certain pH. This pH is defined as the point of zero charge (p.z.c.) (Figure 5). The p.z.c. is a unique value depending on each oxide and an indicator to show an acid or basic property. In the case of TiO_2_, the p.z.c. of rutile is 5.3, and that of anatase is 6.2 (Parfitt, 1976); therefore, TiO_2_ does not show an outstanding acid or basic property but shows almost a neutral property. The concentration of surface hydroxyl groups on TiO_2_ is relatively large−4.9–12.5 nm^−2^ (Boehm, 1971; Westall and Hohl, 1980). After immersion in aqueous solution, this concentration or wettability increases. This large concentration promotes the adsorption of proteins, such as integrin and cytokine.

### Protein Adsorption

**Figure 6:**
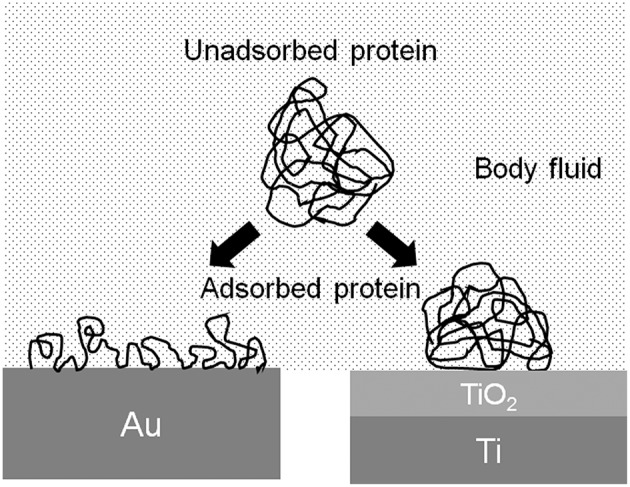
Schematic model of change in the conformation of protein adsorbed on Au and Ti.

The conformation of proteins is changed by the adsorption to the metal surface, because proteins are charged objects. The electrostatic force of proteins to a metal surface is governed by the relative permittivity of the surface oxide film: the larger the relative permittivity, the smaller the electrostatic force. The relative permittivity of TiO_2_ is much larger than those of other oxides, 82.1, and similar to that of water (80.0) (Lide, 2006). Therefore, the conformational change of protein adsorbed on TiO_2_ is possibly small (Figure 6). The adsorption layer of fibrinogen is thicker, but the adsorption amount is smaller on Ti than on Au in aqueous solution (Sundgren et al., 1986a). The electrostatic force on Ti is small, but on Au is large, because Ti is covered by TiO_2_ and Au metal exposes without surface oxide. The change in the conformation of proteins on Ti is smaller than that on Au. Proteins adsorbed on Ti are less susceptible.

### Formation of Calcium Phosphate

The composition and chemical state of surface oxide film vary according to the surrounding environment; while the film is macroscopically stable. A passive film maintains a continuous process of partial dissolution and reprecipitation in the electrolyte from the microscopic viewpoint. In this sense, the surface composition is always changing according to the environment (Kelly, 1982). Ti and Ti alloys easily form calcium phosphates on themselves in a biological environment, and form sulfite and sulfide, especially under cell culture (Hanawa and Ota, 1991, 1992; Healy and Ducheyne, 1992; Serro et al., 1997; Hiromoto et al., 2004). Ti is stabilized after the formation of calcium phosphate in Hanks' solution (Tsutsumi et al., 2009). In addition, calcium and phosphorus are detected at the interface between Ti and bone tissue (Sundgren et al., 1986b; Esposito et al., 1999; Sundell et al., 2017). One of the reasons for the excellent hard-tissue compatibility in Ti is its ability to form calcium phosphate.

## Surface Treatment of Titanium

### Category

**Figure 7:**
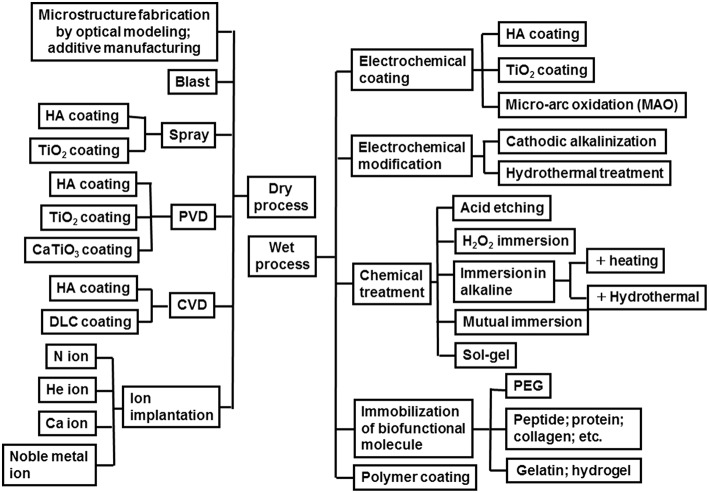
Category of surface finishing and surface treatment of Ti to accelerate bone formation, bone bonding, soft tissue adhesion, wear resistance, antibacterial property and blood compatibility.

**Figure 8:**
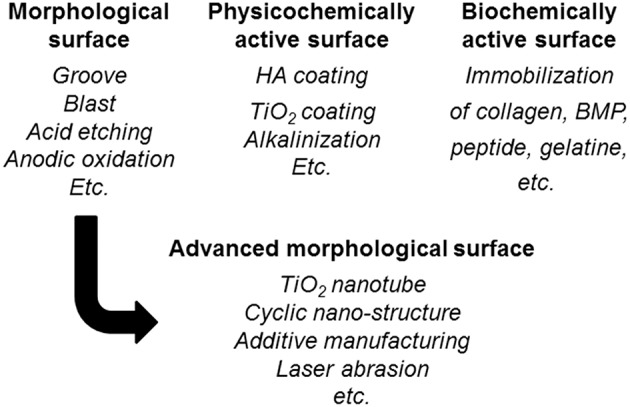
Surface finishing and surface treatment of Ti to accelerate bone formation and bone bonding.

To promote the biocompatibility of Ti and to add biofunction to Ti while retaining the advantage of its mechanical property, surface treatment is necessary. Surface treatment techniques for Ti continue to be reviewed (Brunette et al., 2001; Hanawa, 2009, 2017; Williams, 2011; Ratner et al., 2013; Civantos et al., 2017). Surface treatment techniques for medical applications are categorized in Figure 7, and most of them are commercially viable in the engineering field. However, some of them were originally developed for medical devices. In addition, the major purpose of surface treatments is to accelerate bone formation and bonding. Another category of surface finishing and surface treatment of implants is summarized in Figure 8. Recently, immunomodulatory applications to regenerate tissues have attracted the attention of biomaterials researchers (Lee et al., 2019). As shown in Figure 8, surface treatments and their effects are summarized in the following subsections.

### Control of Surface Morphology and Porous Surface

Surface roughness influences the healing and remodeling process of tissues. Osteoblastic cells adhere well to rough metal surfaces *in vitro* (Rautray et al., 2011). Surface roughness also plays an important role for the differentiation of cells. For example, osteoblast accelerates collagen production and calcification on rough surfaces rather than on smooth surfaces (Keller et al., 1994). The shear bonding force increases with increasing roughness. Influence of surface topography on osseointegration has been studied (Albrektsson and Wennerberg, 2004; Wennerberg and Albrektsson, 2010; Nagasawa et al., 2016; Rupp et al., 2018). The surface roughness of a material is an important factor for bonding of tissues. Mechanical anchoring results from the ingrowth of bone tissue into pores. Even in the case where surface treatment improves the chemical composition, the effects of not only the chemical composition but also the roughness produced simultaneously by the treatment appear in most cases to accelerate bone formation and bone bonding.

**Figure 9:**
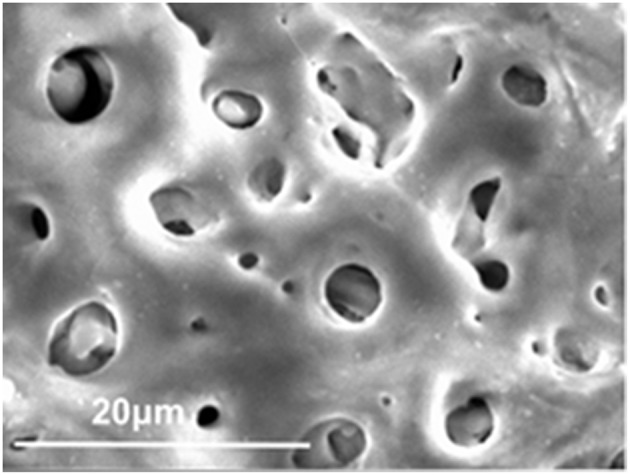
Porous TiO_2_ oxide layer formed on Ti by micro-arc oxidation.

The first surface treatment for biomaterials was the control of surface morphology—that is, the formation of macroscopic grooves or grids. Living tissues become ingrown in holes or pores, and mechanical anchoring is achieved. Plasma spray of Ti and hydroxyapatite (HA) on the stem of artificial joints made of Ti alloys and blast and acid etching in dental implants have been commercialized. Micro-arc oxidation (MAO) or plasma electrolytic oxidation (PEO) to form a connective porous TiO_2_ layer have also been commercialized in dental implants. Bone tissue grows into pores to achieve bonding. A scanning electron micrograph of porous TiO_2_ oxide formed on Ti by MAO is shown in Figure 9.

In the advanced morphology surface fabrication in Figure 8, an evolutional technique of surface morphological control is the formation of TiO_2_ nanotubes promoting cell adhesion and bone formation because of the effect of the nanometer size (Allam et al., 2008; Brammer et al., 2012; Narayanan et al., 2014; Awad et al., 2017). On the other hand, a cyclic nanometer-level structure accelerates bone formation (Shinonaga et al., 2014; Matsugaki et al., 2015). In addition, this structure also accelerates the adhesion and differentiation of a stem cell (Olivares-Navarrete et al., 2010; Chen et al., 2017, 2018). Bone quality is governed not only by bone density but also bone structure orientation (Ishimoto et al., 2013). Grooves oriented to a main stress vector have been designed that control the orientation of the bone structure (Noyama et al., 2013). This technique has been commercialized in a dental implant. Recently, studies to control bacterial adhesion by a cyclic structure at a micrometer level have been increasing in number (Anselme et al., 2010). Nanotopographies have been applied to form antibacterial surfaces (Orapiriyakul et al., 2018; Mas-Moruno et al., 2019).

Three-dimensional additive manufacturing is an effective tool to form the above surface morphology (Wang et al., 2016). Additive-manufactured implants have been clinically applied, and effective ingrowth of bone to porous implants has been observed (Wang et al., 2017; Gao et al., 2018).

### Hydroxyapatite and Oxide Coatings

To form a physicochemical active surface, HA is a main inorganic component of tooth and bone, so a coating of HA has been popular for accelerating bone formation and increasing resistance (Harun et al., 2018). The first technique was plasma spray (Ong and Lucas, 1994), which has been applied to various products. Thereafter, other coating techniques to form HA have been developed. Physical vapor deposition (PVD) in dry processes and electrochemical formation in wet processes are predominant, while a sol-gel technique (Li et al., 1996) and alternate immersion technique (Taguchi et al., 1999) have been developed. In addition, coatings of bioactive glass, tricalcium phosphate (Kitsugi et al., 1996), carbonate apatite (Yamaguchi et al., 2010), and octacalcium phosphate (Lin et al., 2003) with a bone formation ability larger than that of HA have been studied and developed. On the other hand, TiO_2_ and other oxides have been coated on Ti (Umetsu et al., 2013). The surface is simultaneously roughened with a spray coating.

### Surface Modification Layer Formation

**Figure 10:**
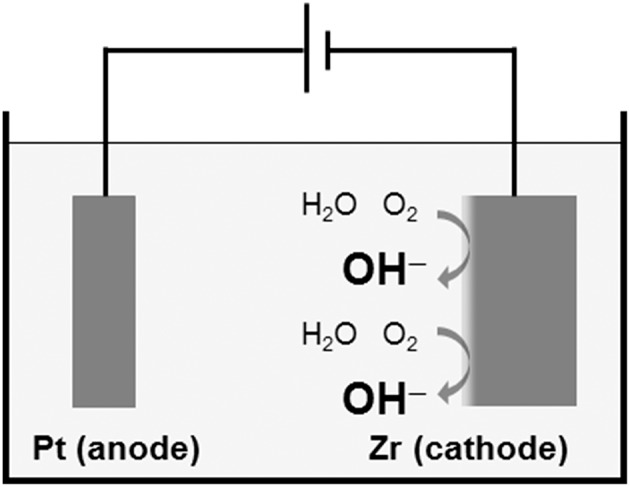
Local alkalinization of Zr surface by cathodic polarization in a supporting electrolyte solution.

Another technique to form a physicochemical active surface has been developed. The Ti surface is activated without coatings of HA and calcium phosphate. This surface is expected to form HA in bone tissue spontaneously. The oldest technique is calcium ion implantation (Hanawa et al., 1993, 1997). On the other hand, when Ti is immersed in an alkaline solution, such as NaOH and KOH, and heated, the surface is alkalinized, and the alkaline component is released to body fluid, followed by HA formation to form bone (Kim et al., 1996). This technique has been commercialized in an artificial hip joint. However, this technique is not effective for Zr, which does not form calcium phosphate on itself. Thus, Zr is cathodically polarized, and the surface of Zr is locally alkalinized, as shown in Figure 10 (Tsutsumi et al., 2010).

### Immobilization of Biofunctional Molecules and Biomolecules

The ideas of improvement of bone formation and of bone bonding by the immobilization of biomolecules involved in bone formation to a metal surface are logical. Such biomolecules as peptides, gelatins, and bone morphogenetic protein (BMP) are immobilized on the Ti surface (Hanawa, 2013). Immobilization of Type I collagen (Morra et al., 2011), fibronectin (Pegueroles et al., 2011), Arg-Gly-Asp (RGD) array peptide (Yamamichi et al., 2008), and BMP (Schliephake et al., 2012) is effective to promote cell spreading and bone formation. Immobilization of biomolecules has also been applied to create antibacterial surfaces (Qin et al., 2018). In the case of electrodeposition of poly(ethylene glycol) (PEG), the PEG-immobilized Ti inhibits protein adsorption, platelet adhesion (Tanaka et al., 2010a), and bacteria (Tanaka et al., 2010b).

The idea that the bone formation of a material's surface becomes active by the immobilization of biomolecules in bone formation is reasonable, and many studies have been conducted. However, to popularize the immobilization of biofunctional molecules widely, it is necessary to ensure the safety, maintenance of quality during storage, and dry-conditioned durability of the immobilized layer. It is difficult for manufacturers to commercialize this technique unless they see value in commercialization. There are many problems with commercializing the immobilized materials, although it is easy to show good results in basic research.

### Cleaning and Hydrophilic Treatment

Surface contamination prevents bone formation and bone bonding in dental implants (Ueno et al., 2012). Instruments for optical activation treatments, such as ultraviolet irradiation and plasma irradiation, are available. Surface contamination is removed, and surface hydroxyl groups appear on the Ti surface in these optical activation treatments. The bone formation ability of a material is related to its wettability (Yamamoto et al., 2012). Surface characteristics of Ti implant have been reviewed elsewhere (Rupp et al., 2018).

## Summary and Perspective

Ti is the most biocompatible material among metals. Unfortunately, the underlying mechanism still has not been elucidated completely. Research and development have been focused on surface treatments to improve bone formation and bone bonding, leaving behind the understanding of the mechanism. However, the mechanism of the biocompatibility of Ti is gradually being understood with the research on surface-treated materials. Ti is the most bioactive material among metals, but it is less active than bioactive ceramics. The elucidation of the relevant mechanism can accelerate the development of optimal surfaces. The surface treatment techniques introduced in this review make it possible to apply metals to a scaffold in regenerative medicine or tissue engineering.

## Author Contributions

The author confirms being the sole contributor of this work and has approved it for publication.

### Conflict of Interest Statement

The author declares that the research was conducted in the absence of any commercial or financial relationships that could be construed as a potential conflict of interest.
